# Genomic features and computational identification of human microRNAs under long-range developmental regulation

**DOI:** 10.1186/1471-2164-12-270

**Published:** 2011-05-27

**Authors:** Ying Sheng, Christopher Previti

**Affiliations:** 1 Computational Biology Unit, Bergen Center for Computational Science, and Sars International Centre for Marine Molecular Biology, University of Bergen, Bergen, 5008, Norway; 2 Department of Medical Genetics, Oslo University Hospital, Oslo, 0407, Norway

## Abstract

**Background:**

Recent functional studies have demonstrated that many microRNAs (miRNAs) are expressed by RNA polymerase II in a specific spatiotemporal manner during the development of organisms and play a key role in cell-lineage decisions and morphogenesis. They are therefore functionally related to a number of key protein coding developmental genes, that form genomic regulatory blocks (GRBs) with arrays of highly conserved non-coding elements (HCNEs) functioning as long-range enhancers that collaboratively regulate the expression of their target genes. Given this functional similarity as well as recent zebrafish transgenesis assays showing that the miR-9 family is indeed regulated by HCNEs with enhancer activity, we hypothesized that this type of miRNA regulation is prevalent. In this paper, we therefore systematically investigate the regulatory landscape around conserved self-transcribed miRNAs (ST miRNAs), with their own known or computationally inferred promoters, by analyzing the hallmarks of GRB target genes. These include not only the density of HCNEs in their vicinity but also the presence of large CpG islands (CGIs) and distinct patterns of histone modification marks associated with developmental genes.

**Results:**

Our results show that a subset of the conserved ST miRNAs we studied shares properties similar to those of protein-coding GRB target genes: they are located in regions of significantly higher HCNE/enhancer binding density and are more likely to be associated with CGIs. Furthermore, their putative promoters have both activating as well as silencing histone modification marks during development and differentiation. Based on these results we used both an elevated HCNE density in the genomic vicinity as well as the presence of a bivalent promoter to identify 29 putative GRB target miRNAs/miRNA clusters, over two-thirds of which are known to play a role during development and differentiation. Furthermore these predictions include miRNAs of the miR-9 family, which are the only experimentally verified GRB target miRNAs.

**Conclusions:**

A subset of the conserved miRNA loci we investigated exhibits typical characteristics of GRB target genes, which may partially explain their complex expression profiles during development.

## Background

MicroRNAs (miRNAs) are small RNAs (~ 22 nt) found in plants, animals, viruses and at least one unicellular organism (*Chlamydomonas reinhardtii*) [1]. They function by binding to target sites in 3'' UTRs of messenger RNAs (mRNAs) to repress translation or mediate mRNA degradation, although alternative modes of action have been reported recently, such as direct transcriptional silencing of *POLR3D *by miR-320 [2]. In animals, the majority of mature miRNAs are synthesized in two processing steps: first, the primary miRNA transcript (pri-miRNA) is cut by the nuclear RNase III enzyme *Drosha *and its cofactor *DGCR8 *in the nucleus, generating precursor miRNAs (pre-miRNAs). These are subsequently exported to the cytoplasm via the nuclear transport receptor *exportin-5 *and the co-factor *RanGTP*, where they are cleaved by the RNase III enzyme Dicer into a double stranded RNA of ~22nt. The strand with the less stable 5'' hydrogen bonding is usually selected as the mature miRNA, although both strands can be functional. It is assumed that the pri-miRNAs of most intergenic miRNAs are transcribed independently by RNA polymerase II (Pol II) [3], while most intragenic miRNAs are co-transcribed with their host genes. Given the similarity with class II protein-coding genes, their expression may be controlled through a variety of shared regulation pathways. However, few pri-miRNAs have been characterized experimentally to date [3-8], making the localization of their promoters challenging and one of the prime reasons we are only beginning to understand the mechanism by which their expression is regulated. Recently, several studies attempted to predict the primary transcripts of pri-miRNAs by exploring transcription factor binding sites (TFBSs), expressed sequence tags (ESTs), transcription start site (TSS) predictions, and further, complementary genomic data [9-15]. Based on these studies, the length of pri-miRNAs was estimated to range from several hundred to several thousand nucleotides. These studies are useful references to estimate the scale of pri-miRNAs as well as the location of their promoter, in order to investigate miRNA transcriptional regulation.

miRNAs can be expressed in a tissue- and stage-specific manner during development and that they can play key roles in lineage decisions of progenitor cells and organogenesis (reviewed in [16]). Enhancer-mediated long-range regulation is an important mechanism for controlling gene expression during development and has recently been shown to affect miRNAs as well [17]. Many of these enhancers are highly conserved non-coding elements (HCNEs) that collaboratively regulate the specific expression of their respective target genes [18-24]. It has previously been demonstrated that both HCNEs and their target genes are preserved within synteny blocks in vertebrates and insects during evolution, which has served as the foundation for the concept of the "genomic regulatory block" (GRB). GRBs are functional regulatory units that consist of HCNEs, genes regulated by HCNEs ("target genes") as well as "unrelated" genes ("bystander genes"). Both HCNEs and target genes have coevolved in order to maintain their functional association, while bystander genes can be lost through the time.

Further investigation of the general transcriptional initiation properties of genes in GRBs has shown that the promoters of GRB target genes share common features that can be used to distinguish them from the promoters of bystander genes, which are genes that may be close to, or even harbor, HCNEs but are not under their regulation. Target genes are generally associated with long CpG islands (CGIs) that are not limited to the 5' end of the genes, but also occur in introns or internal exons of the gene [25] and coincide with genomic regions bound by repressor Polycomb group proteins [26]. In addition, they have a higher number and wider spacing of alternative TSSs, and a distinct composition of TFBSs in their core/proximal promoters [25]. Furthermore, many target gene promoters belong to the class of "bivalent promoters" that display a distinct pattern of both activating and repressing histone modifications in embryonic stem (ES) cell lines [25], which may allow them to be turned on or off quickly during organogenesis [27,28]. The analysis of the mir-9 miRNA family members (*dre-mir-9-5 *and ) *dre-mir-9-1*) in zebrafish has shown that they are regulated by the same type of enhancers as protein-coding GRB target genes [17]. These miRNA target genes are both embedded in areas of conserved synteny throughout vertebrates and co-localize with a number of HCNEs that function as long-range enhancers controlling their expression. As a result, they show highly stage- and tissue-specific expression in dorsal telencephalon at 24 h post fertilization, while the expression pattern of the neighboring bystander genes is far less specific. The inspection of other miRNAs in the Ancora genome browser [29] clearly reveals further examples of miRNAs that fall within regions of high HCNE density, such as *hsa-mir-124-2*, indicating that this type of regulation may be prevalent.

Based on their functional similarity and the common transcriptional mechanisms they employ, we hypothesize that miRNAs with complex spatiotemporal expression patterns may be regulated in the same manner as protein-coding GRB target genes and that they share the following genomic properties:

- a genomic neighborhood with a high HCNE density

- a bivalent promoter [28] during development and differentiation

- large CGIs spanning both the promoter and the gene body

In our work we show that both a subset of conserved self-transcribed miRNAs (ST miRNAs) as well as a set of experimentally validated GRB target miRNAs [17] demonstrate these properties. But our ability to use these features for predicting novel GRB target miRNAs was limited, since more than half of the ST miRNAs in our dataset do not have primary transcript predictions and we were therefore not able to accurately compute CpG island-related features for them. As a result, we used an elevated HCNE density in the genomic vicinity as well as the presence of a bivalent promoter to then identify 29 putative GRB target miRNAs/miRNA clusters, over two-thirds of which are known to play a role during development and differentiation. Furthermore these predictions include miRNAs of the miR-9 family, which are the only experimentally verified GRB target miRNA genes.

## Results and Discussion

### Defining self-transcribed miRNAs and the extent of their pri-mRNAs

Based on the assumption that some miRNAs have co-evolved with their cis-acting regulatory elements, in this study, we focused on conserved human self-transcribed miRNAs (ST miRNAs), which are assumed to be transcribed from their own promoters and have an ortholog in at least one other vertebrate species (Additional file 1, Table S1). These requirements are consistent with the work describing the original definition of a GRB [17] as well as the subsequent analysis of the features of GRB target genes [25]. In general, ST miRNAs include all conserved human intergenic miRNAs except those transcribed by RNA polymerase III (Pol III) [30], as well as known cases of intronic miRNAs whose fate after whole-genome duplication in fish indicates that they have their own promoters, independent of their host genes (see Methods).

**Figure 1:**
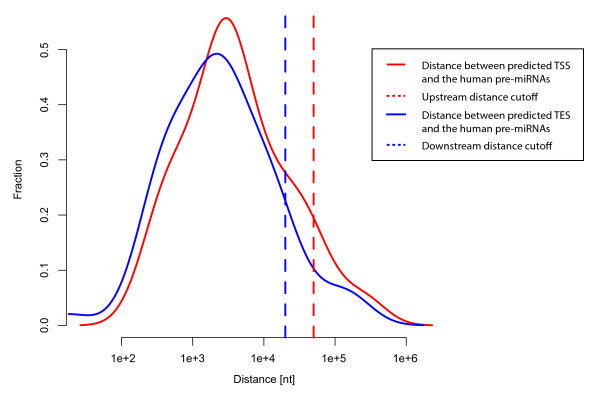
**Density distribution of distances between predicted TSSs/TESs and human pre-miRNAs**. The corresponding dashed lines indicate the cutoffs used to define the TSSs and TESs in the analysis, whose distance distributions are indicated by the red and blue curves, respectively.

Since proximal miRNAs can appear in genomic clusters that share the same promoter and are transcribed as a single transcript, we clustered ST miRNAs according to their genomic vicinity (see Methods) and analyzed only one promoter per cluster,

The annotation of the pri-miRNAs we used in this study was the result of a combinatorial approach by Saini et al. [14] that employed predicted and experimental evidence to identify both transcription start and end sites. One of the most important reasons for choosing this dataset was the fact that they considered a larger range of possible miRNA sizes, thereby avoiding biases introduced by excluding very long transcripts. We estimated the general scale of pri-miRNAs based on these data, where the TSS is within 50 kb upstream of pre-miRNAs and the transcription end is within 20 kb downstream of the pre-miRNAs. This approach was sensitive enough to include around 84% of the pri-miRNAs in the initial dataset (see Figure 1 and Methods).

### Enhancer density

#### HCNEs

**Figure 2:**
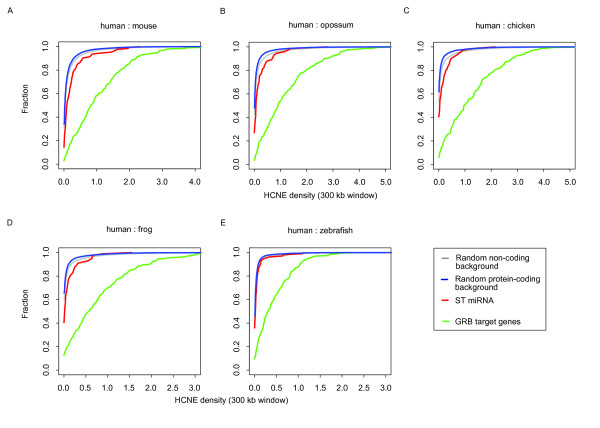
**The enrichment of HCNEs around conserved human ST miRNAs (including ST miRNAs overlapping with GRBs)**. Figure 2 shows the cumulative curves of HCNE density in five lineage comparisons. The lineages compared are indicated at the top of each figure. The HCNE density was calculated based on a 300 kb window centered on a region of interest, which is either a ST miRNA, a randomly selected coding/non-coding region (control sets) or a GRB target gene. The x-axis shows the percentage of base pairs in HCNEs within the 300 kb window (HCNE density). The fraction of 300 kb windows we analyzed is shown in the y-axis. The red curve shows the HCNE density of the conserved human ST miRNAs, while the grey, blue and green curves show the HCNE density of the non-coding and protein-coding control sets as well as the set of GRB target genes, respectively. Conserved human ST miRNAs are therefore more likely to be located in regions with higher HCNE density than would be expected by chance.

HCNEs represent putative enhancers of gene expression, whose frequency rises strongly around the conserved developmental genes they usually target [17,22]. We therefore first investigated whether this applied to conserved ST miRNAs as well, by comparing the HCNE density in a 300 kb window centered on the ST miRNA with random coding and non-coding genomic regions with similar gene density (see Methods). In a few cases (~8%), the location of the ST miRNA search window overlapped with a previously defined GRB making it difficult to identify the actual target gene, which could either be the miRNA, the annotated GRB target gene, or both. For this reason, we compared the HCNE densities both including and excluding cases in which the ST miRNA search window overlapped with a previously defined GRB. As shown in Table 1, the HCNE density was significantly higher around ST miRNAs than the random coding and non-coding regions in both comparisons, a trend that is independent of the lineages compared (*p*-value ≤ 0.05, two-sided bootstrapped version of the Kolmogorov-Smirnov test: see Table 1, Figure 2 as well as Additional files 2 and 3). Thus, in support of our original hypothesis, we can conclude that HCNEs are highly over-represented in the genomic vicinity of conserved human ST miRNAs. The HCNE density was also compared between the conserved ST miRNAs and the GRB target genes annotated in Akalin et al. [25]. We found that the HCNE density was significantly lower for the ST miRNAs (*p*-value ≤ 0.05, two-sided bootstrapped version of the Kolmogorov-Smirnov test: see Table 1, Figure 2 as well as Additional files 2 and 3). This is due to the fact that the GRB target genes were annotated based on their high HCNE density [25], which was not a prerequisite for constructing our dataset of ST miRNAs.

**Table 1:** HCNE density comparison

	Comparison between human conserved ST miRNAs and human random coding regions		Comparison between human conserved ST miRNAs and human random non-coding regions		Comparison between human conserved ST miRNAs and human GRB target genes	
	
Lineage comparison	*p*-value (all regions)	*p*-value (excluding regions overlapping GRBs)	*p*-value (all regions)	*p*-value (excluding regions overlapping GRBs)	*p*-value (all regions)	*p*-value (excluding conserved human ST miRNAs overlapping GRBs)
human: mouse	0	0	0	0	0	0
human: dog	0	0	0	0	0	0
human: opossum	0	0	1.0e-04	0	0	0
human: platypus	0	0	0	2.0e-04	0	0
human: chicken	0	0	0	0	0	0
human: frog	0	0	0	0	0	0
human: zebrafish	4.0e-03	8.0e-03	1.8e-03	4.0e-04	0	0

The comparisons represented by each column were performed after selecting HCNEs and ST miRNAs conserved between the lineages shown in the left column (see Methods). The *p*-values were computed using the two-sided bootstrapped version of the Kolmogorov-Smirnov test. All p-values lower than 1.0e-20 were set to 0.

#### Experimentally verified enhancers

**Figure 3:**
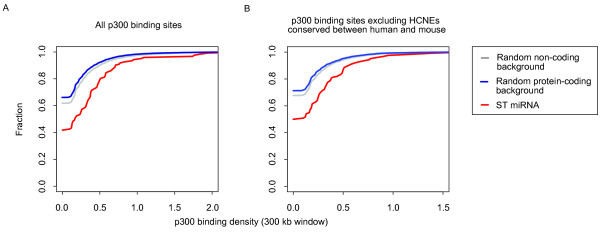
**The enrichment of p300 binding sites around mouse orthologs of human ST miRNAs**. Figure 3 shows the cumulative curves of the enhancer enrichment analysis using all p300 binding sites (A) and using only p300 binding sites that do not overlap HCNEs conserved between human and mouse (percentage of identity ≥ 98% and length of HCNE ≥ 50 bp) (B). These results indicate that the mouse orthologs of human ST miRNAs are more likely to be located in regions with significantly higher p300 binding site density than the control set of protein coding and non-coding regions.

In addition to HCNEs, we utilized experimental data describing the location of the transcriptional co-activator p300 to analyze the enhancer density around ST miRNAs. This transcription factor is a ubiquitous component of enhancer-associated protein assemblies. It co-localizes with active enhancers and plays a critical role during embryonic development [31-36]. Visel et al. [36] mapped the genome-wide binding of p300 in forebrain, midbrain and limb tissue of developing mouse embryos using ChIP-seq technology. This generated ~5000 p300 binding regions which are associated with active enhancers. Since conservation criteria did not play a role in determining these p300 binding regions, they represent a dataset of experimentally verified enhancers that is completely independent of the HCNEs, allowing us to test our hypothesis in an unbiased manner. We then compared the p300 binding density around ST miRNAs that are conserved between mouse and human (mouse:human) with that of mouse coding and non-coding regions using the same method as the previous HCNE density analysis and found that the density of p300 binding sites around mouse:human ST miRNAs was indeed significantly higher than in the control sets (*p-*value < 1e-20 for both comparisons, two-sided bootstrapped version of the Kolmogorov-Smirnov test) (Figure 3).

These results are independent of HCNE density, since the same trend was still present when we performed the analysis excluding p300 binding sites that overlap with mouse orthologs of our HCNEs (*p*-value < 1e-20 for both comparisons, two-sided bootstrapped version of the Kolmogorov-Smirnov test) (Figure 3, Additional file 4 and Table S2).

### Comparison between enhancer enriched and enhancer poor ST miRNAs

As the density of HCNEs/p300 binding around conserved ST miRNAs is significantly higher than in random non-coding and protein coding regions and there are conserved ST miRNAs that are known developmental regulators similar to GRB target genes, we wanted to see whether there was an association between the individual features of GRB target genes and HCNE/p300 densities. We again limited the analysis to ST miRNAs conserved between human and mouse in order to be able to incorporate the p300 binding data.

We then compared the features of GRB target genes between the HCNE/p300 enriched and HCNE/p300 poor miRNAs excluding intragenic ST miRNAs due to the uncertainty in associating genomic features with the ST miRNA or the host gene.

#### Identification and analysis of bivalent ST miRNA promoters

The genome-wide mapping of chromatin states by detection of histone marks revealed regions carrying both the H3K4me3 and H3K27me3 histone modifications. Although these so-called bivalent domains [27] are rare within the genome, they are over-represented at the promoters of key developmental genes (bivalent promoters) in embryonic stem cells [28] and were suggested to mediate activation and repression of expression of the genes during lineage commitment by maintaining genes in a poised status [27]. Furthermore, bivalent promoters represent useful marks for the annotation of both protein-coding and miRNA GRB target genes, since around 70% of them had a bivalent promoter in mouse ES cells, compared to only 13% of the bystander genes [25], and the expression of several lineage-specific miRNAs is correlated with the presence of this mark at their promoters [28].

Both human [37] and mouse bivalent domains [28] were subsequently mapped to the putative promoter regions of HCNE enriched/poor ST miRNAs and p300 enriched/poor ST miRNAs, respectively (see Methods). We found that 24% (15/63) of the HCNE enriched ST miRNAs are associated with bivalent domains within 50 kb upstream in human ES cells, compared to only 8% (5/62) of HCNE poor ST miRNAs (*p*-value = 0.03, one side Fisher''s exact test). However, bivalent domains are less likely to be associated with p300 enriched ST miRNAs compared to those of p300 poor ST miRNAs (10/62 versus 19/61). This difference could be explained by the fact that the data on histone modifications and p300 binding are from different tissues/developmental stages, since the histone modification data are from V6.5 ES cells, hybrid ES cells, Neural Progenitor cells (NPCs) and primary Embryonic Fibroblasts (MEFs) obtained at embryonic day (E) 13.5 [28], while the p300 data is from embryonic forebrain, midbrain and limb tissue of mouse embryos at E 11.5 [36]. As enhancers and histone modifications are both tissue- and stage-specific, it is possible that the p300 data and bivalent domains we used in the analysis regulate transcription in distinct developmental contexts. Furthermore, the bivalent promoters are associated with promoters "poised" for transcription [27] while the p300 marks of active enhancers are expected to co-occur with actively transcribed target genes [38], so they are unlikely to co-occur in the same tissue/stage for a given gene. Fortunately, the detection of HCNEs does not rely on tissue or developmental stages, but instead is able to detect putative enhancers in all tissues from all stages. Therefore, the analysis of HCNE enriched/poor ST miRNAs is stage/tissue-independent and thus less likely to be biased. For this reason, in the following analysis we only compared features between HCNE enriched and HCNE poor ST miRNAs.

#### Analysis of CpG islands associated with ST miRNAs

GRB target genes are often associated with higher ratios between CGI length and transcript length (CpG-to-gene ratio), and the overlapping CGIs map not only to the promoter, as in most other genes, but also introns, internal exons, and in some cases, even cover the entire target gene [25]. It was shown recently that some of these CGIs coincide within genomic regions bound by repressive Polycomb Group proteins (PcG-proteins) [26]. A recent study also found that 21 human miRNAs co-localized with multiple CGIs within their 10 kb flanking regions, and that 25 pre-miRNAs were completely embedded in CGIs [12]. In addition, Juan et al. [39] showed that the expression of miR-199/214 might be regulated by PcG-proteins during skeletal muscle cell differentiation. In another example shown by Wang et al. miR-29 is repressed by NF-kappaB acting through YY1 and the PcG-proteins [40]. Based on these findings, we compared the CpG-to-gene ratio associated with HCNE enriched and HCNE poor ST miRNAs as well as the gene sets used in the previous GRB target gene study by Akalin et al [25] (see Methods).

The results show that the CpG-to-gene ratio of the HCNE enriched ST miRNAs is significantly higher than those of bystander, transcription factor and CGI-associated genes, while there is no significant difference in the comparison with the GRB target genes (Table 2). Furthermore, we find no significant differences in the CpG-to-gene ratios between HCNE poor ST miRNAs and bystander genes or other transcription factors. In addition, the CpG-to-gene ratios of HCNE poor ST miRNAs is significantly lower when compared to GRB target genes/other CGI genes. This indicates that the distribution of CpG-to-gene ratios of HCNE enriched ST miRNAs is more similar to GRB target genes than that of HCNE poor ST miRNAs. However, we did not find significant differences in the CpG-to-gene ratios between HCNE enriched ST miRNAs and HCNE poor ST miRNAs, which could be explained by the small sample size. Nevertheless, there is still a common trend showing that HCNE enriched ST miRNAs have higher CpG-to-gene ratios than HCNE poor ST miRNAs, since their median CpG-to-gene ratio is fivefold higher (Table 2).

**Table 2:** Comparison of CpG-to-gene ratios between different gene sets

	HCNE enriched miRNAs	HCNE poor miRNAs	Known GRB target genes	Known bystander genes	Other transcription factors	Other CpG island genes
Median CpG-to-gene ratio	0.1703	0.0238	0.2032	0.0100	0.0339	0.0280
*p*-values:						
HCNE enriched miRNAs	-	0.2931	0.1185	**2.7450e-4**	**0.0124**	**2.7800e-3**
HCNE poor miRNAs	0.2931	-	**0.0158**	0.1846	0.0966	**1.4200e-3**

Significant differences in the median CpG-to-gene ratio between the gene sets were determined using the two-sided bootstrapped version of the Kolmogorov-Smirnov test. Significant *p*-values (<0.05) are shown in bold. The first row indicates the median CpG-to-gene ratio for each gene set and the second and third rows contain the *p*-values of the comparisons between the CpG-to-gene ratios of the HCNE enriched or HCNE poor ST miRNAs and other control gene sets.

### GRB target gene features can identify ST miRNAs subject to long-range regulation

**Figure 4:**
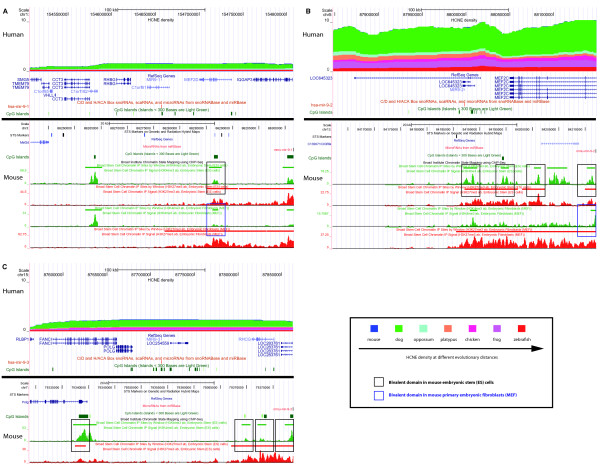
**Case study of the miR-9 family**. UCSC Genome Browser screen shots of the miRNAs, *hsa-mir-9-1 *(A), *hsa-mir-9-2 *(B) and *hsa-mir-9-3 *(C) as well as their orthologs in the mouse genome. The screen shots of the human genome display information on CGIs, neighboring protein-coding genes as well as the level of HCNE density in different lineage comparisons. The screen shots of the mouse orthologs display information regarding bivalent domains (marked by rectangles). The color of the rectangle indicates the cell type the bivalent domain was detected in.

The miR-9 family of miRNAs is a known, experimentally verified GRB target [17] and therefore a prime example for illustrating how the genomic features we analyzed could serve to annotate miRNAs under long-range regulation. This family is specifically expressed in brain and affects the fate of ES cell-derived neural precursor cells differentiating along the glial or neuronal pathways [41]. The expression of the miR-9 family of genes is dynamically regulated [42] during differentiation and development, and the human miR-9 family has three members: *hsa-mir-9-1*, *-2*, and *-3*. In the human genome, *hsa-mir-9-2 *and *hsa-mir-9-3 *are intergenic miRNAs, and *hsa-mir-9-1 *is found in the second intron of *C1orf61*. Since the zebrafish ortholog of *hsa-mir-9-1 *is located in an intergenic region, it qualifies as a ST miRNA based on our definition. Moreover, *hsa-mir-9-1 *and *hsa-mir-9-2 *are paralogs that were most likely separated by the whole-genome duplication at the root of jawed vertebrates [43-45], which suggests that their common ancestor was intronic, but that the host gene is not required for their transcription [17]. Therefore, all miR-9 family members can be classified as ST miRNAs and are likely to have their own promoters. A detailed examination of their genomic environments showed that all of them share the features of GRB target genes (Figure 4), since they map to genomic regions with high HCNE densities, the putative promoters of their mouse orthologs map to bivalent domains and they are all associated with several proximal CGIs.

We further examined the annotation of each gene within the investigated region around the miRNAs. Since there is no other putative GRB target gene within the region of *hsa-mir-9-3*, we conclude that *hsa-mir-9-3 *is most likely the only target of long-range enhancers in that region. In the neighborhood of *hsa-mir-9-1 *and *hsa-mir-9-2*, we find myocyte enhancer factors *MEF2D *and *MEF2C*, respectively. Both of these genes are transcription factors that play a role in myogenesis and are therefore plausible GRB target gene candidates. However, it was shown that two regions with enhancer activity located ~10 kb downstream of dre-mir-9-1, the zebrafish ortholog of *hsa-mir-9-1*, and ~100 kb downstream of *dre-mir-9-5*, the zebrafish ortholog of *hsa-mir-9-2*, gave the reporter gene an expression pattern similar to that of zebrafish miR-9, but not the zebrafish myocyte enhancer factors [17]. Thus, it may very well be that both miRNAs and the myocyte enhancer factors are regulated by distinct elements in the regions, and that there is a boundary between the two, similar to that of neighboring GRBs targeting *PAX6 *and *WT1 *genes [46]. In summary, the genomic features of regions around members of the miR-9 family display characteristics equivalent to those of protein-coding GRB target genes, lending further support to the use of these features for predicting novel miRNA targets of long-range regulation.

### Genome-wide identification of putative GRB target miRNAs

Taken together, the results of our analyses show that HCNE enriched ST miRNA genes are more likely to be associated with the features of GRB target genes than HCNE poor ST miRNA genes, which leads us to believe that we can utilize them for the prediction of GRB target miRNAs.

In order to identify GRB target miRNA genes on a genome-wide scale, we selected all HCNE enriched ST miRNA genes across different lineage comparisons, which were associated with bivalent domains in at least one developmental cell type (Table 3). Our predictions included 29 ST miRNA genes/miRNA gene clusters, 19 of which have known functions in development (Additional file 5 and Table S3) as well as the miR-9 family, which are the validated GRB target miRNAs. We did not use CGI-related features in the prediction process, since most of the ST miRNAs in our dataset do not have primary transcript predictions. We were therefore unable to accurately assign CGIs to them. Nevertheless, the majority of them are associated with at least one CGI within the estimated pri-miRNAs (regions within 50 kb up- and 20 kb downstream of the pre-miRNAs).

**Table 3:** Annotation of ST miRNA candidates under putative long-range developmental regulation

Name	Intergenic/Intragenic	**Cell type of bivalent promoter **^**1**^	**Potential alternative GRB target gene **^**2**^	**CGIs **^**3**^
*hsa-mir-196b*	Intergenic	mES, MEF	HOXA cluster	0
*hsa-mir-132~212*	Intergenic	hES, mES	-	1
*hsa-mir-196a-2*	Intergenic	MEF	HOXC cluster	2
***hsa-mir-9-1***	**Intron of *C1orf61***	**mES, MEF**	**-**	**-**
***hsa-mir-9-3***	**Intergenic**	**hES, mES**	**-**	**6**
***hsa-mir-9-2***	**Intergenic**	**hES**	***MEF2C***	**8**
*hsa-mir-10a*	Intergenic	hES, mES, MEF	HOXB cluster	1
*hsa-mir-196a-1*	Intergenic	hES	HOXB cluster	6
*hsa-mir-137*	Intergenic	hES	-	2
*hsa-mir-375*	Intergenic	hES, mES	*FEV*, *INN*	2
*hsa-mir-124-2*	Intergenic	hES, mES	-	3
*hsa-mir-542~450b*	Intergenic	hES, mES	-	3
*hsa-mir-219-2*	intergenic	hES, mES	-	1
*hsa-mir-708*	Intergenic	hES	-	1
*hsa-mir-365-2*	Intergenic	hES, mES	-	3
*hsa-mir-193a*	Intergenic	hES, mES	-	3
*hsa-mir-129-1*	Intergenic	hES, mES, MEF	-	2
*hsa-mir-129-2*	intergenic	hES, mES, MEF	-	3
*hsa-mir-124-1*	Intergenic	hES, mES, MEF	-	3
*hsa-mir-146b*	Intergenic	hES	*NFKB2*	0
*hsa-mir-370*	Intergenic	hES	-	0
*hsa-mir-124-3*	Intergenic	mES, MEF	-	3
*hsa-mir-17~92a-1*	Intergenic	MEF	-	1
*hsa-mir-182~183*	Intergenic	hES, MEF	-	4
*hsa-mir-1-1*	Intron of *C20orf166*	mES	*GATA5*	-
*hsa-mir-133a-2*	Intron of *C20orf166*	mES	*GATA5*	-
*hsa-mir-203*	Intergenic	mES, MEF	-	4
*hsa-mir-16-1~15a*	Intron of *DLEU2*	mES	-	-
*hsa-let-7a-3~let-7b*	Intergenic	mES	*WNT7B*	3

List of conserved human ST miRNAs that have been annotated as targets of putative long-range regulation during development and differentiation. The mir-9 family of miRNAs is highlighted since it contains know examples of GRB target miRNAs that were captured using our two prediction features: 1) localization in regions of high HCNE density and 2) association with a bivalent promoter.

^1^The cell type in which the promoter of the miRNA is predicted to be a bivalent promoter. hES and mES represent embryonic stem cells of human and mouse, respectively. MEFs are mouse embryonic fibroblasts and NPCs are mouse neural progenitor cells.

^2^Further annotated GRB target genes within the 300kb region that was analyzed. The annotation of the GRB target genes was performed as described in the Methods section.

^3^Number of CpG islands overlapping with pri-miRNAs. The pri-miRNA is defined as the region 50 kb upstream to 20 kb downstream of the pre-miRNAs. If the defined pri-miRNA overlapped known protein-coding genes (the gene itself plus 1 kb up- and downstream of it), it was truncated to exclude the overlapping gene (see Methods). We did not count CGIs for intragenic miRNAs (denoted as '-').

## Conclusions

While it has previously been demonstrated that a subset of miRNA genes are under the same type of extreme long-range transcriptional regulation typical for developmental transcription factors [17], we were now able to identify further putative GRB target miRNAs on a genome-wide scale based on the same characteristics defining protein-coding GRB target genes. These features are their localizations in regions with a high HCNE density and the fact that many of them have "bivalent" promoters before or during differentiation.

Furthermore, the majority of our predictions is known to play a role during development and is associated with one or more CGIs that are distributed along the miRNA primary transcript, indicating that these ST miRNAs may be subject to the same type of Polycomb-mediated repression seen in protein coding developmental genes.

Our results provide an important new resource for the analysis of miRNA regulation and greatly increase the number of miRNA genes under putative long-range transcriptional control, indicating that many miRNAs are subject to strict regulatory constraints whenever they are required to establish complex spatiotemporal developmental patterns.

## Methods

### Assemblies and annotation

The following genome assemblies were used: human NCBI 36, mouse NCBI 37 and mouse NCBI 36, chicken v2.1, *Xenopus tropicalis *v4.1 (US DoE JOINT genome Institute), zebrafish Zv7 (The Wellcome Trust Sanger Institute). Genomic coordinates and sequences of known miRNAs were obtained from miRBase (Version 12)[1] and the genomic coordinates of HCNEs were made available from the Ancora database ([29]; http://ancora.genereg.net; Additional file 6, Table S4). CGI annotations, Broad Institute Chromatin State Mapping using ChiP-Seq and genomic sequences were extracted from the UCSC Genome Browser Database ([47]; http://genome.ucsc.edu) and gene annotations for human, mouse and zebrafish were obtained from Ensembl ([48]; http://www.ensembl.org; version 52) using Biomart ([49]; http://www.biomart.org). In addition, because of the limited annotation of the zebrafish genome, we also included zebrafish orthologs of human genes predicted based on the similarity of exonic sequences and gene structures between zebrafish and human genomes [50]. The list of putative GRB target genes was constructed by inspecting HCNE density peaks, tracing the teleost gene/HCNE synteny, and analyzing functional gene annotation [25]. GRBs are human-zebrafish synteny blocks overlapping with GRB target genes. Synteny blocks were defined through the joined zebrafish to human high scoring net alignments downloaded from the UCSC Genome Browser [47].

### miRNA classification

Human and zebrafish miRNAs were classified into intergenic and intragenic miRNAs based on the overlap of the pre-miRNA with known protein coding genes (at least 1 bp overlapping). Our ST miRNAs included both human intergenic miRNAs and human intragenic miRNAs that were intergenic in zebrafish genome, since these are likely to be self-transcribed. We removed the Pol III-transcribed miRNAs mentioned by Borchert et al [30] and subsequently retrieved orthologs of human ST miRNAs conserved at different evolutionary distances. For each lineage comparison we clustered human ST miRNAs into genomic clusters if they were less than 10 kb apart.

### Identification of orthologous miRNAs

Orthologs of human ST pre-miRNAs were identified in seven other species (mouse, dog, opossum, platypus, chicken, frog and zebrafish). We used BLAST with default parameters, with the exception of word size, which was set to six, to compare all known pre-miRNAs and mature miRNAs between all species. The best reciprocal BLAST hits highlighted miRNA pairs that were chosen as putative orthologs. In cases where we could not find orthologs at the pre-miRNA level, we used the mature miRNA instead. For precursor orthologs, we required that the length of the aligned region was equal to or greater than 56% of the query length and that the gap-size was no greater than 10.

For orthologs identified via the mature miRNA, we required that the length of the aligned region was equal to or greater than 80% of the query length and that the number of non-matching bases is was no greater than three. Furthermore, the first two to eight bases had to be conserved.

### Estimating the scale of pri-miRNAs

We used the annotation produced by Saini et al. [14] to examine the distance between putative miRNA transcription start sites/transcription ends and their associated pre-miRNAs. We found that 93% of the TSSs were within 50 kb upstream of the pre-miRNAs and 90% of the miRNA transcription ends lay within 20 kb downstream of the pre-miRNAs (Figure 1). These results were used to define the general span of miRNA transcripts and extend the analysis window accordingly.

For the analysis of miRNAs with primary transcript predictions, i.e. the study of CGIs and bivalent promoters, we applied a search window that was the same size as the predictions from the study mentioned above [14]. Otherwise, the search window was 50 kb upstream and 20 kb downstream of the pre-miRNAs in the CGI analysis and only the region 50 kb upstream of the pre-miRNA in the bivalent promoter analysis. If the search window overlapped known/annotated protein-coding gene regions (the gene itself plus 1 kb up- and downstream of it) it was truncated to exclude the overlapping gene(s).

### Analysis of enhancer density

Enhancer (HCNE or p300 binding site) densities were scanned using a 300 kb sliding window as described in Engström et al.[29]. For the density of the region around a sequence feature of interest (TSS for protein-coding genes), we used the HCNE density value in a 300 kb window centered on the feature.

We constructed 10000 non-coding/200 protein-coding control sets by randomly selecting the same number of coding and non-coding regions in the same genome as the conserved ST miRNAs. They had distributions of length, intergenic/intragenic ratio and gene density equal to that of the conserved ST miRNAs. For the comparison with GRB target genes, we used the whole set of GRB target genes.

### Separation of ST miRNAs based on HCNE or p300 density

In the analysis of p300 density, we first ordered the human:mouse conserved ST miRNAs by human:mouse HCNE densities and mouse:human conserved ST miRNAs by p300 binding densities. Then we evenly divided human and mouse conserved ST miRNAs into two groups. The top half is located in regions enriched in HCNE/p300 binding (HCNE/p300 enriched miRNAs), whereas the remaining ST miRNAs were classified as being located in regions that are depleted in HCNE/p300 binding (HCNE/p300 poor miRNAs).

For predicting ST miRNAs that are located in regions of high HCNE density on a genome-wide scale, we ordered the conserved ST miRNAs by their HCNE densities, using HCNEs conserved between human and one of the seven vertebrate species shown in Table S4 and dividing them into HCNE enriched and HCNE poor ST miRNAs in the same way as mentioned above.

### Identification of bivalent domains and bivalent promoters

Bivalent domains are regions that are enriched in both H3K4me3 and H3K27me3 histone modifications at the same developmental stage and the same tissue. We only predicted bivalent promoters for intergenic miRNAs since it was impossible to distinguish whether the features were associated with the ST miRNA or the host gene. Mikkelsen et al. [28] made the mouse histone modification information available and the human histone modification information was originally from Pan et al. [37]. We predicted bivalent promoters of protein-coding genes and miRNAs using their respective definitions.

### Comparison of CpG island features

We compared the CpG island features between HCNE enriched and HCNE poor miRNAs as well as protein-coding GRB target genes, bystander genes and two other control sets, that were comprised of known transcription factors and CGI-associated genes. These gene sets were taken from the previous study of GRBs by Akalin et al. [25]. Contrary to this prior work, we only used the CpG-to-gene ratio in our analyses and not CGI length and count, since the length of the putative primary ST miRNAs is much shorter than that of protein-coding genes (*p*-value = 1e-03; one-sided Wilcoxon test). Furthermore, we focused only on miRNAs that have primary transcript predictions in order to ensure a high level of accuracy.

## List of abbreviations

CGI: CpG island; ES cell: Embryonic stem cell; EST: Expressed sequence tags; GRB: Genomic regulatory block; HCNE: Highly conserved non-coding element; MEF: Primary embryonic fibrobalst; miRNA: microRNA; NPC: Neural progenitor cell; Pol II: RNA Polymerase II; Pol III: RNA Polymerase III; ST miRNA: Self-transcribed miRNA; TFBS: Transcription factor binding site; TSS: Transcription start site

## Authors' contributions

YS and CP conceived and designed the experiments. YS performed the experiments. YS and CP wrote the manuscript. Both authors critically read and approved the final version.

## Supplementary Material

Additional file 1 **Number of human ST miRNAs conserved in different vertebrate lineages**. The number of human ST miRNAs investigated in our study that is conserved between human and different vertebrate lineages. Click here for file

Additional file 2 **Enrichment of HCNEs around human ST miRNAs, conserved in dog (A) and platypus (B)**. As in Figure 2, the graphs are cumulative HCNE density curves for conserved human ST miRNAs. The HCNEs shown here are conserved in dog (A) and platypus (B) as indicated at the top of each figure pair. In keeping with our results, conserved human ST miRNAs are more likely to be located in regions with higher HCNE density than would be expected by chance and this association extends to the entire vertebrate lineage. Click here for file

Additional file 3 **Enrichment of HCNEs around conserved human ST miRNAs (excluding ST miRNAs overlapping with GRBs)**. Each sub-figure shows the cumulative HCNE density curves for conserved human ST miRNAs in distinct lineage comparisons, but this time, excluding ST miRNAs overlapping with known GRBs. These results confirm that conserved human ST miRNAs are also more likely to be located in regions with higher HCNE density than would be expected by chance, independently of their association with any known GRBs and are therefore most likely the actual target of long-range regulation. Click here for file

Additional file 4 **Number of p300 binding regions overlapping mouse HCNEs**. Total number of p300 binding regions and those overlapping with mouse HCNEs (with percentage of identity larger than 98% and length longer than 50 bp) in three mouse embryonic tissues. Click here for file

Additional file 5 **Functional annotation of miRNAs under putative long-range regulation**. Functional information regarding the miRNA candidates we annotated as being under putative long-range regulation. Click here for file

Additional file 6 **The percentage of identity and length cutoffs of HCNEs**. The lineage comparisons, percentage of identity and length cut-offs used to determine the HCNEs in our study. Click here for file
