# Supplementary material for: Genomic features and computational identification of human microRNAs under long-range developmental regulation
Source: BMC Genomics. 2011 May 27;12:270. doi: 10.1186/1471-2164-12-270 (PMC3123655; doi:10.1186/1471-2164-12-270)
Supplement: Additional file 1 — Number of human ST miRNAs conserved in different vertebrate lineages. The number of human ST miRNAs investigated in our study that is conserved between human and different vertebrate lineages. [file 1471-2164-12-270-S1.DOC]

**Table S1. Number of conserved human ST miRNAs present in different vertebrate lineages**

| Lineage comparison: | human : mouse | human : dog | human : opossum | human : platypus | human : chicken | human : frog | human : zebrafish |
| --- | --- | --- | --- | --- | --- | --- | --- |
| intergenic ST miRNAs | 82 | 60 | 46 | 56 | 53 | 59 | 62 |
| intronic ST miRNAs* | 43 | 37 | 28 | 39 | 41 | 40 | 44 |
| Total | 125 | 97 | 74 | 95 | 94 | 99 | 106 |

*transcriptional independence from the host gene inferred from the fate of the miRNA after whole-genome duplication in fish genomes, where at least one copy no longer lies within a gene.
