# Supplementary material for: Genomic features and computational identification of human microRNAs under long-range developmental regulation
Source: BMC Genomics. 2011 May 27;12:270. doi: 10.1186/1471-2164-12-270 (PMC3123655; doi:10.1186/1471-2164-12-270)
Supplement: Additional file 2 — Enrichment of HCNEs around human ST miRNAs, conserved in dog (A) and platypus (B). As in Figure 2, the graphs are cumulative HCNE density curves for conserved human ST miRNAs. The HCNEs shown here are conserved in dog (A) and platypus (B) as indicated at the top of each figure pair. In keeping with our results, conserved human ST miRNAs are more likely to be located in regions with higher HCNE density than would be expected by chance and this association extends to the entire vertebrate lineage. [file 1471-2164-12-270-S2.PDF]

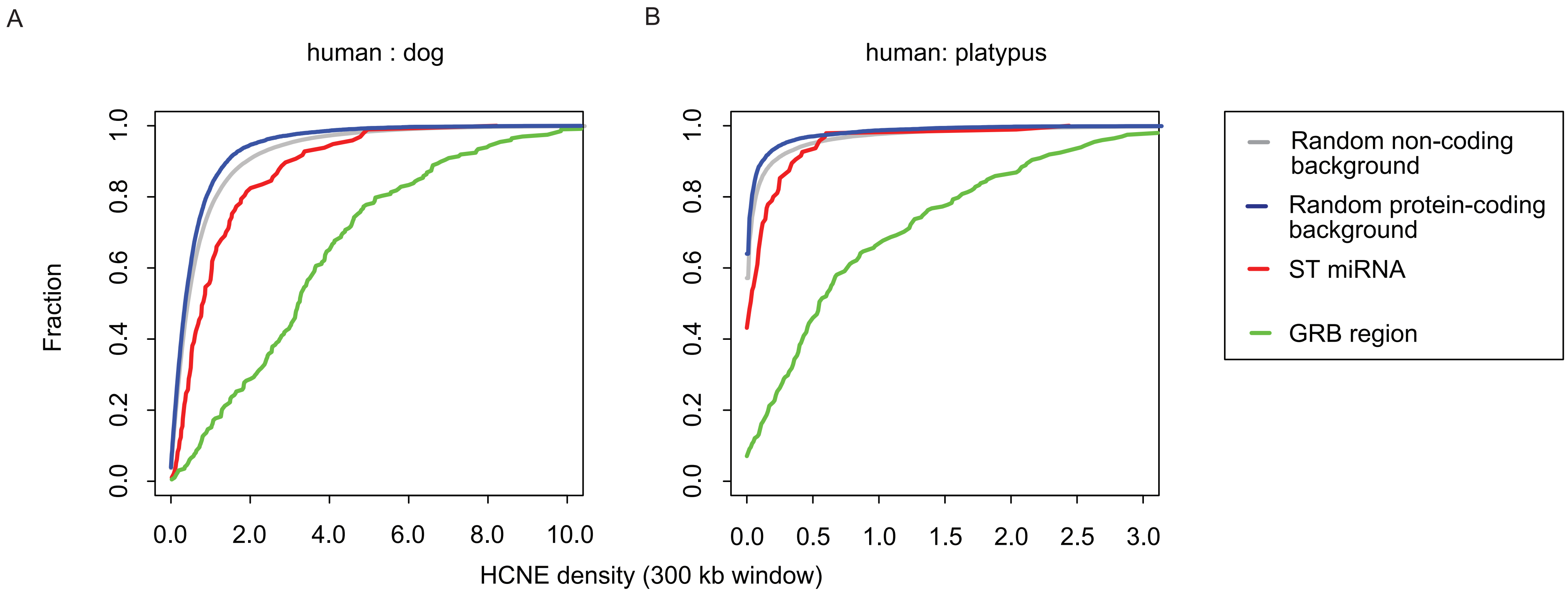

A
human : dog
B
human: platypus
Fraction
0.0
0.2
0.4
0.6
0.8
1.0
0.0
2.0
4.0
6.0
8.0
10.0
0.0
0.5
1.0
1.5
2.0
2.5
3.0
HCNE density (300 kb window)
Random non-coding background
Random protein-coding background
ST miRNA
GRB region
