# Supplementary material for: Genomic features and computational identification of human microRNAs under long-range developmental regulation
Source: BMC Genomics. 2011 May 27;12:270. doi: 10.1186/1471-2164-12-270 (PMC3123655; doi:10.1186/1471-2164-12-270)
Supplement: Additional file 3 — Enrichment of HCNEs around conserved human ST miRNAs (excluding ST miRNAs overlapping with GRBs). Each sub-figure shows the cumulative HCNE density curves for conserved human ST miRNAs in distinct lineage comparisons, but this time, excluding ST miRNAs overlapping with known GRBs. These results confirm that conserved human ST miRNAs are also more likely to be located in regions with higher HCNE density than would be expected by chance, independently of their association with any known GRBs and are therefore most likely the actual target of long-range regulation. [file 1471-2164-12-270-S3.PDF]

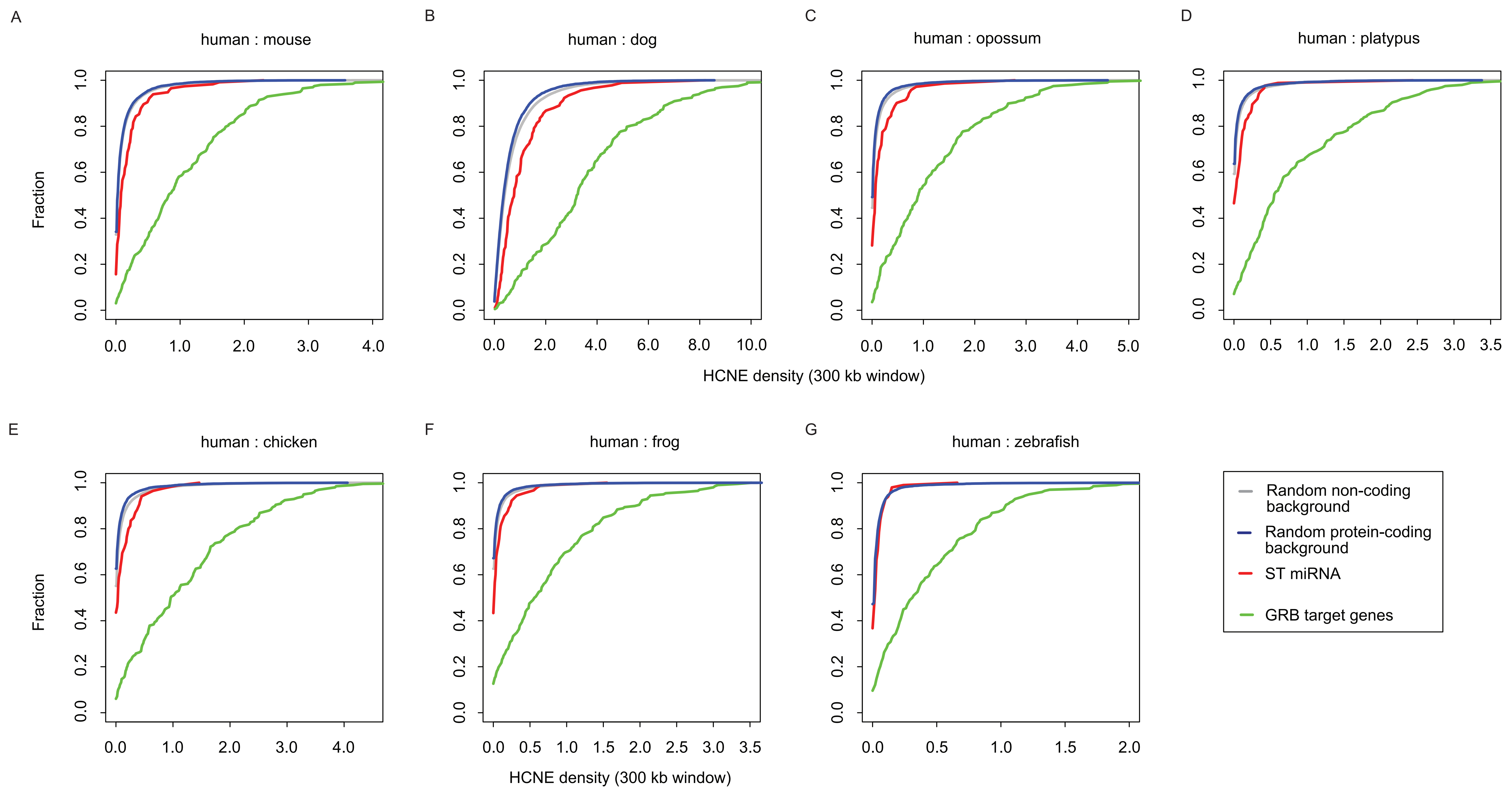

A
human : mouse
B
human : dog
C
human : opossum
D
human : platypus
Fraction
HCNE density (300 kb window)
E
human : chicken
F
human : frog
G
human : zebrafish
Fraction
HCNE density (300 kb window)
Random non-coding background
Random protein-coding background
ST miRNA
GRB target genes
