# Supplementary material for: Genomic features and computational identification of human microRNAs under long-range developmental regulation
Source: BMC Genomics. 2011 May 27;12:270. doi: 10.1186/1471-2164-12-270 (PMC3123655; doi:10.1186/1471-2164-12-270)
Supplement: Additional file 4 — Number of p300 binding regions overlapping mouse HCNEs. Total number of p300 binding regions and those overlapping with mouse HCNEs (with percentage of identity larger than 98% and length longer than 50 bp) in three mouse embryonic tissues. [file 1471-2164-12-270-S4.DOC]

**Table S2. Number of p300 binding regions overlapping mouse HCNEs.**

|  | Mouse embryonic forebrain | Mouse embryonic midbrain | Mouse embryonic limb |
| --- | --- | --- | --- |
| Number of overlapping p300 binding regions | 974 | 188 | 601 |
| Total number of p300 binding regions in the examined tissue | 2454 | 562 | 2106 |
