# Supplementary material for: Genomic features and computational identification of human microRNAs under long-range developmental regulation
Source: BMC Genomics. 2011 May 27;12:270. doi: 10.1186/1471-2164-12-270 (PMC3123655; doi:10.1186/1471-2164-12-270)
Supplement: Additional file 5 — Functional annotation of miRNAs under putative long-range regulation. Functional information regarding the miRNA candidates we annotated as being under putative long-range regulation. [file 1471-2164-12-270-S5.DOC]

**Table S3. Available functional information for the miRNA candidates under putative long-range regulation.**

| Name | Functional annotation |
| --- | --- |
| *hsa-mir-196b* | Associated with regulation of Hox genes , tail regeneration , anti-viral defense |
| *hsa-mir-132~212* | Associated with regulation of synaptic structure and function , ischemic preconditioning-induced tolerance , inflammation , experience-dependent neuronal plasticity , Gonadotropin-releasing hormone stimulated biosynthetic response , biological clock , Alzheimer’s disease and cancer . |
| *hsa-mir-196a-2* | See function of *hsa-mir-196b* |
| *hsa-mir-9-1* | Specifically expressed in brain, can significantly affect the cell fate of ES cell-derived neural precursor cells differentiating along the glial or neuronal pathways . |
| *hsa-mir-9-3* | See function of *hsa-mir-9-1* |
| *hsa-mir-9-2* | See function of *hsa-mir-9-1* |
| *hsa-mir-10a* | miR-10 and Hox gene co-expression during the development and several Hox genes are targets of miR-10 |
| *hsa-mir-196a-1* | See function of *hsa-mir-196b* |
| *hsa-mir-137* | Associated with early erythroid commitment , lymph node metastasis of colon cancer , induce differentiation of adult mouse neural stem cells, mouse oligodendroglioma-derived stem cells and human glioblastoma multiforme-derived stem cells and induce glioblastoma multiforme cell cycle arrest , down-regulates micropthalmia-associated transcription factor in melanoma cell lines |
| *hsa-mir-375* | Associated with pancreatic development, regulation of insulin secretion and exocytosis (reviewed in ) and cancer . |
| *hsa-mir-124-2* | Specifically expressed in brain, plays an important role in ES cell-derived neural precursor cell differentiation . Another study further identified the SCP1 gene, critical to inducing neurogenesis, as a direct target of miR-124. It is also associated with Pancreatic islet development. |
| *hsa-mir-542~450b* | Unknown function; miR-542 is associated with pathogenesis of endometriosis |
| *hsa-mir-219-2* | Unknown function; overexpression or knockdown of miR-219 leads to embryonic defects in zebrafish development . |
| *hsa-mir-708* | Unknown function; associated with childhood acute lymphoblastic leukemia |
| *hsa-mir-365-2* | Unknown function; associated with endometriosis |
| *hsa-mir-193a* | Unknown function |
| *hsa-mir-129-1* | Associated with retina development , differentiation of hematopoietic stem cells , undifferentiated gastric cancer , bladder cancer , colorectal cancer and human esophageal squamous cell carcinoma |
| *hsa-mir-129-2* | See function of *hsa-mir-129-1* |
| *hsa-mir-124-1* | See function of *hsa-mir-124-2* |
| *hsa-mir-146b* | Associated with immaturity of neonatal immune system , acute LPS-induced inflammation , innate immune response and cancer . |
| *hsa-mir-370* | Associated with lipid metabolism, hepatic ischemia/reperfusion injury and ischemic preconditioning and cancer . |
| *hsa-mir-124-3* | See function of *hsa-mir-124-2* |
| *hsa-mir-17~92a-1* | Unknown function: associated with cancer |
| *hsa-mir-182~183* | Associated with lung tumorigenesis |
| *hsa-mir-1-1* | Specifically expressed in cardiac and skeletal muscle. They promote mesoderm formation from ES cells and repress nonmuscle gene expression and cell fate during cell-lineage commitment |
| *hsa-mir-133a-2* | See function of *hsa-mir-1-1* |
| *hsa-mir-203* | Associated with epidermal differentiation |
| *hsa-mir-16-1~15a* | Associated with chronic lymphocytic leukemia |
| *hsa-let-7a-3~let-7b* | Unknown function: associated with cancer |
