# Supplementary material for: Genomic features and computational identification of human microRNAs under long-range developmental regulation
Source: BMC Genomics. 2011 May 27;12:270. doi: 10.1186/1471-2164-12-270 (PMC3123655; doi:10.1186/1471-2164-12-270)
Supplement: Additional file 6 — The percentage of identity and length cutoffs of HCNEs. The lineage comparisons, percentage of identity and length cut-offs used to determine the HCNEs in our study. [file 1471-2164-12-270-S6.DOC]

**Table S4. The percentage of identity and length cutoffs of HCNEs.**

| HCNEs | Percentage of identity cutoff | Length cutoff |
| --- | --- | --- |
| human:mouse | 98% | 50 |
| human:dog | 96% | 50 |
| human:opossum | 96% | 50 |
| human:platypus | 96% | 50 |
| human:chicken | 90% | 50 |
| human:frog | 80% | 50 |
| human:zebrafish | 70% | 50 |
